# WHAT CAN DUAL-TASK WALKING AND TAPPING TELL US ABOUT SUBJECTIVE COGNITIVE DECLINE? AN FNIRS STUDY

**DOI:** 10.1093/geroni/igac059.1309

**Published:** 2022-12-20

**Authors:** Talia Salzman, Hannah Perreault, Farah Farhat, Diana Tobon Vallejo, Sarah Fraser

**Affiliations:** University of Ottawa, Ottawa, Ontario, Canada; University of Ottawa, Ottawa, Ontario, Canada; University of Ottawa, Ottawa, Ontario, Canada; Universidad de Medellin, Medellin, Antioquia, Colombia; University of Ottawa, Ottawa, Ontario, Canada

## Abstract

Older adults who pass standard cognitive tests but report subjective cognitive decline (SCD) may be identifying early changes in cognition at a stage when intervening can prevent further declines. Changes may be subtle highlighting the need for novel approaches, such as divided attention tasks, to distinguish between those with and without SCD. This pilot study examined 15 older women (9 SCD, 6 non-SCD) completing dual-task walking and tapping. Brain (cerebral oxygenation) and behavioural (gait and tap speed, accuracy, and vocal response) measures were assessed during single and dual-tasks. Older adults with SCD were marginally less accurate during dual-task tapping (p < .06) and had greater cerebral oxygenation than the non-SCD group (p = .01). SCD did not moderate gait speed from single to dual-task while non-SCD did (p = .02). Findings suggest that challenging dual-task paradigms may help identify different behavioural and brain activity markers of SCD and intervention targets.

